# Sporadic late onset nemaline myopathy with concurrent dermatological symptoms responding to immunosuppressive treatment

**DOI:** 10.1186/s12883-023-03283-7

**Published:** 2023-06-16

**Authors:** Anirban Nandy, Hatice Tankisi, Anne Bruun Krøigård, Maiken Glud Dalager, Marie Skov Hvidbjerg, Henrik Daa Schrøder, Izabella Obál

**Affiliations:** 1 Department of Neurology, Aalborg University Hospital, Ladegaardsgade 5, Aalborg, 9000 Denmark; 2 Department of Clinical Neurophysiology, Aarhus University Hospital, Palle Juul-Jensens Boulevard 99, Aarhus, 8200 Denmark; 3 Department of Pathology, Odense University Hospital, J. B. Winsløws Vej 4, Odense, 5000 Denmark; 4 Department of Dermatology, Aalborg University Hospital, Hobrovej 18-22, Aalborg, 9000 Denmark; 5 Department of Clinical Genetics, Aalborg University Hospital, Ladegaardsgade 5, Aalborg, 9000 Denmark

**Keywords:** Muscle disease, Nemaline, SLONM, Skin manifestations, Autoimmunity

## Abstract

**Background:**

Sporadic late onset nemaline myopathy is a rare, progressive muscle disease, presenting in adulthood, mainly affecting proximal limb and bulbar muscles. Muscle biopsies show characteristic nemaline rods. The putative mechanism is considered immune-related. Other manifestations aside from neuromuscular symptoms have not been described previously.

**Case presentation:**

We present a case with atypical sporadic late onset nemaline myopathy (SLONM) of a non-HIV, non-MGUS subtype, where skin manifestations preceded neuromuscular symptoms, and a residual thymus with the histology of thymic follicular hyperplasia was detected during the diagnostic workup. Thorough dermatological investigations could not explain the skin presentations. Muscle biopsy revealed variation in fiber diameter, ragged-red and COX-negative fibers associated with discrete fibrosis. Electron microscopy detected atrophic muscle fibres with disorganization of the myofibrils, nemaline rods and abnormal mitochondria. Single-fiber EMG suggested signs of a neuromuscular transmission defect, EMG showed signs of myopathy. Analyses of antibodies associated with myasthenia gravis were negative. The patient showed improvement after intravenous immunoglobulin treatment regarding both the skin and the muscle symptoms.

**Conclusions:**

Our case highlights the heterogeneity of SLONM with its varied spectrum of presentation. A unique combination of dermatological symptoms and SLONM could be seen with skin lesions as primary presenting symptoms. An association can be considered between the different manifestations, presumably based on immune etiology, where immunosuppressive therapy has been beneficial.

## Background

Sporadic late onset nemaline myopathy is a rare, acquired muscle disease presenting in adulthood, predominantly characterized by proximal limb weakness with frequent bulbar, axial, limb muscle and respiratory involvement [1] [2] [3]. Muscle biopsies show pathological accumulation of nemaline rods in muscle fibers. SLONM is frequently associated with monoclonal gammopathy of unknown significance (MGUS) and HIV infection. [2] [3] [4]. The detection of MGUS and HIV positivity is important because the choice and effectiveness of treatment can be affected by the subtype of SLONM [2] [3]. The treatment regimen includes immunosuppressive therapy, whereas, especially in MGUS-SLONM, chemotherapy (melphalan) and autologous stem cell transplantation may be considered [2] [3] [4] [5].

## Case Presentation

We describe the case of a 59-year-old woman, with previously known hypertension, osteoporosis and slight thrombocytopenia, who presented with recurrent rashes and blisters on the extremities and the trunk, approximately one year before developing neurological symptoms. Dermatological investigations have not revealed any other specific dermatosis, besides an episode of urticaria. The main causes of blistering diseases, including porphyria, have been excluded. Genetic examination for acute intermittent porphyria was negative. The patient was referred to our neuromuscular outpatient clinic because of progressive head drop, speech and swallowing difficulties, without clear daily variation. Neurological examination revealed a pronounced head drop with neck extension muscle strength of 4 on the MRC scale, bilateral ptosis, reduced facial mimic, mild dysarthria and dysphagia. Fatiguability could not be observed. Sensorimotor examinations and reflexes were normal. Scapular winging or myotonia was not observed.

Aggravation of the symptoms could be seen during the follow up with a neck extension muscle strength of 3 on the MRC scale and more significant swallowing difficulties requiring adjustments in food intake. The skin lesions had been present in the year prior to the onset of muscle weakness but could also be detected continuously after.

**Fig. 1:**
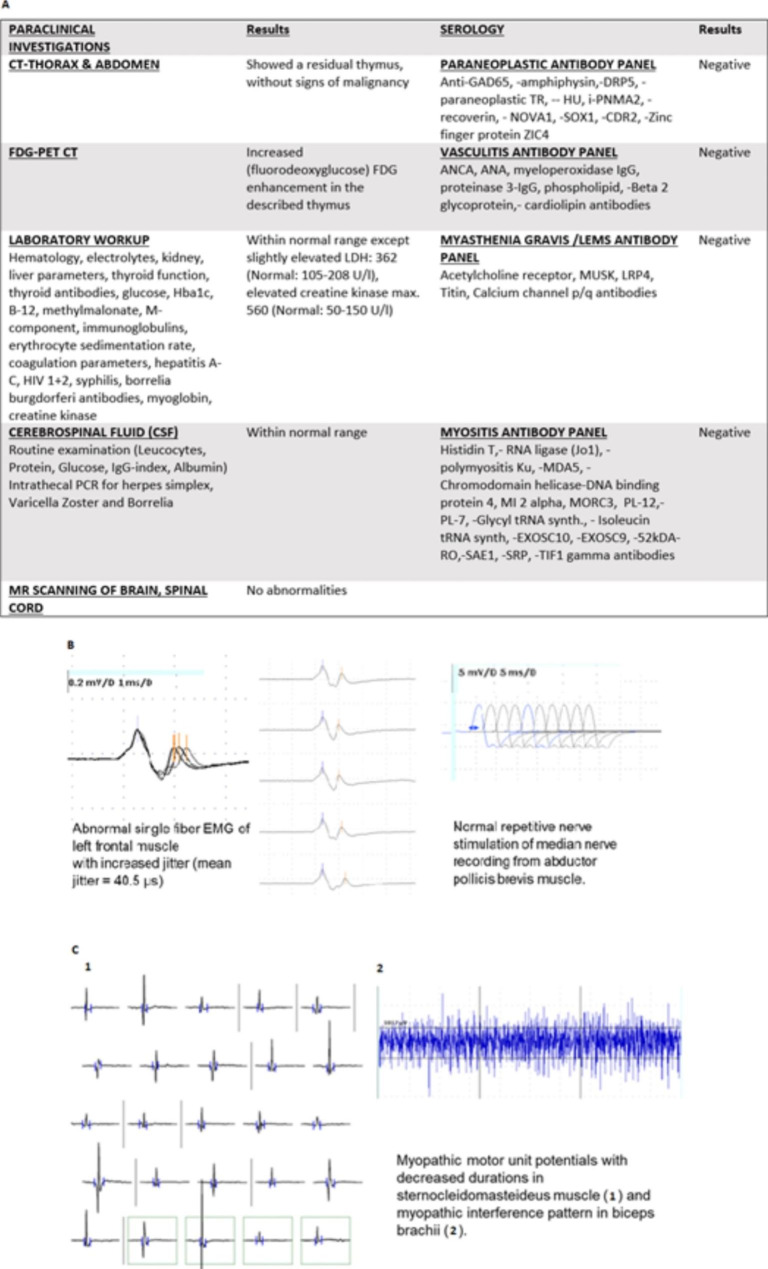
Diagnostic workup. **A**. The list of all the paraclinical examinations. **B.** Single fiber EMG of the left frontal muscle with increased jitter (mean jitter: 40.5 µs (normal value: mean 20.6 ± 3.6 SD). Repetitive nerve stimulation of the median nerve, recording from the abductor pollicis brevis muscle. **C**. Myopathic motor unit potentials with decreased durations from the m. sternocleidomastoideus and myopathic interference pattern in m. biceps brachii

An extensive workup focusing on a presumed myopathy, myasthenia, malignancy and associated paraneoplastic syndrome was carried out. Figure 1 A. Biochemical analysis showed a slightly increased creatine kinase, myoglobin and LDH level. Thoracal CT and PET CT scanning showed a residual thymus with FDG enhancement. Other results were normal.

Quantitative electromyography (EMG) showed signs of myopathy in the sternocleidomastoid, brachial biceps, medial vastus and anterior tibial muscles. There were only fibrillations and positive sharp waves in the biceps brachii. Repetitive nerve stimulation was normal both in proximal and distal muscles. Single fibre EMG (sfEMG) of the frontalis muscle suggested a neuromuscular transmission defect. Figure 1B, C. Motor and sensory nerve conduction studies were normal.

**Fig. 2:**
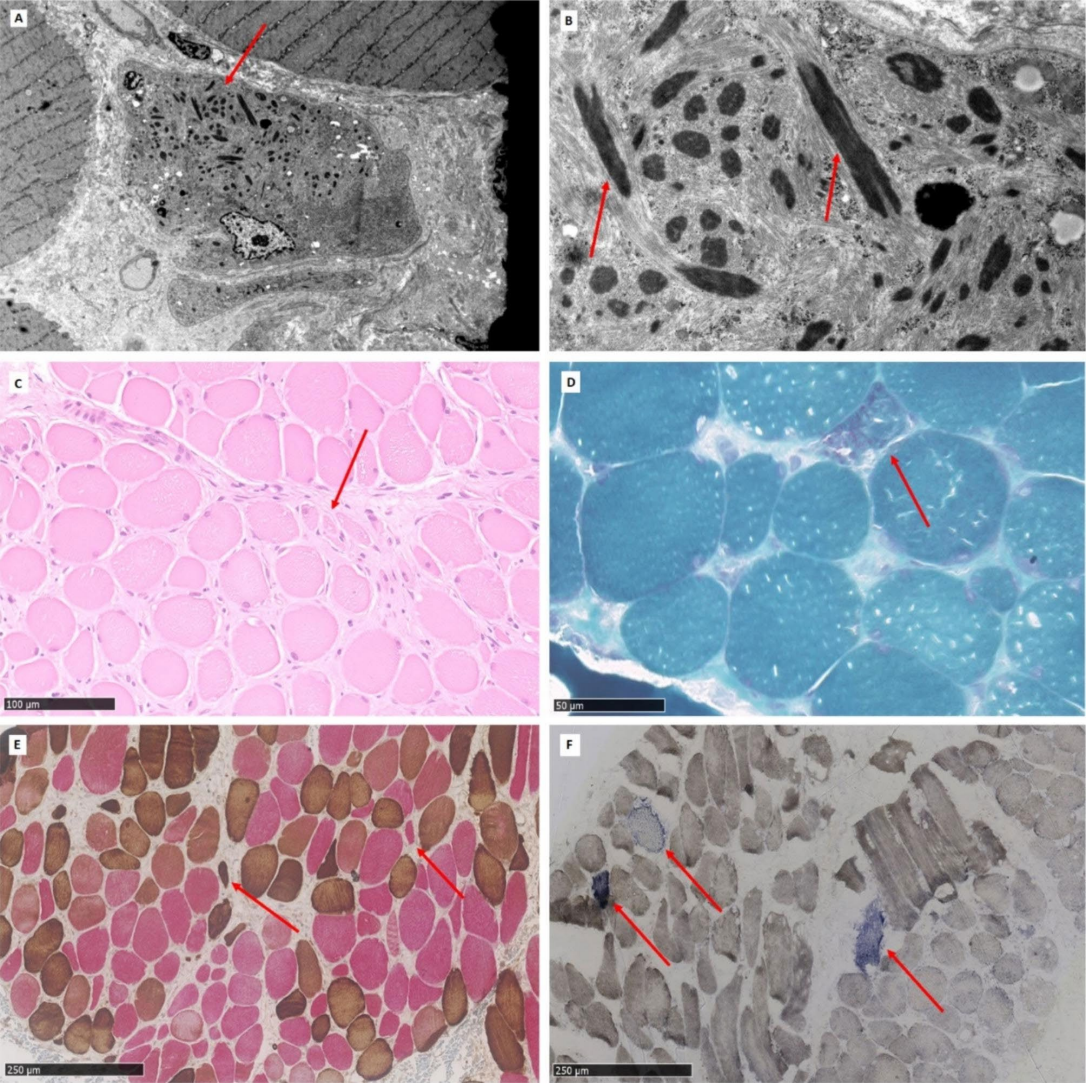
Muscle biopsy. **A**. Electron micrograph of skeletal muscle fibers. In the center an atrophic fiber with activated nucleus is seen. The abnormal myofibrillar structure with aggregates of Z-disc material, i.e. the nemaline rods can be seen as “dark material” (arrows). **B.** Electron micrograph of a skeletal muscle fiber revealing disorganization of the myofibrils. Nemaline rods consisting of aggregates of Z-disc material are seen (arrows). No cores were identified. No infiltrates of lymphocytes or plasma cells were detected. **C**. Hematoxylin and eosin staining, revealing the presence of round and angular atrophic fibers (arrow) as well as increased amount of interstitial collagen **(D)** Gomori trichrome staining revealing a singel ragged red fibre (arrow). **(E)** Double **s**taining for slow (red) and fast (brown) myosin reveals predominance of type 1 fibres. Atrophy can be observed among both type 1 (red) and type 2 (brown) fibres (arrows). **(F)** Staining for COX/succinate dehydrogenase shows a few COX negative fibres (arrows)

Muscle biopsies from the m. splenius capitis were taken with a 3-month difference. The age of the patient was 59 at the time of the biopsies. Light microscopy of the muscle biopsy revealed fibre diameter variations with the presence of round and angular atrophic fibres and vacuolisation. Figure 2 C-F. An increased amount of interstitial collagen was seen, and type I predominance could be observed. Atrophy was visible among both type 1 and type 2 fibres. No central nuclei, but regenerating fibre could be detected. No necrosis was visible. Gomori trichrome staining showed a few ragged red fibres. Figure 2D. A few COX-negative fibres were also detected in double staining for COX/succinate dehydrogenase. Figure 2 F. The presence of rods could not be excluded. Infiltration of T cells or other inflammatory features, such as upregulation of MHC I or MAC could not be detected. The cytoplasmatic lipid content was normal.

Electron microscopy revealed atrophic muscle fibre with disorganization of the myofibrils and aggregates of Z-disc material in the form of numerous nemaline rods, as well as abnormal mitochondria. The conclusion was nemaline rod myopathy. Figure 2 A, B.

As mitochondrial diseases can present with similar symptoms, we aimed to investigate the possibility of mitochondrial etiology. A metabolic examination of the biopsies was done, which showed normal respiratory chain enzyme activity. Moreover, a PCR analysis of the whole mitochondrial genome was carried out from muscle cell DNA, extracted from a part of the muscle biopsy sample. The analysis showed a normal result corresponding to the patient`s age, mitochondrial gene mutations could not be found.

An NGS analysis of 256 genes associated with myopathies, including those responsible for congenital nemaline myopathy, showed a negative result. Table 1.

**Table 1:** Shows the NGS myopathy panel used including the genes for congenital nemaline myopathy

NGS analysis- myopathy panel:
ABHD5, ACADS, ACADVL, ACTA1, ACTN3, ACVR1, AGK, AGL, AGRN, ALG13, ALG14, ALG2, ANO5, AR, ATP2A1, B3GALNT2, B3GNT1, B4GALNT1, BAG3, BICD2, BIN1,C10orf2, CACNA1A, CACNA1S, CAPN3, CASQ1, CAV3, CCDC78, CFL2, CHAT, CHKB, CHRNA1, CHRNB1, CHRND, CHRNE, CHRNG, CKM, CLCN1, CMYA5, CNBP, CNTN1, COL12A1, COL6A1, COL6A2, COL6A3, COLQ, COX15, CPT2, CRYAB, DAG1, DCST2, DES, DMD, DNAJB6, DNM2, DOK7, DOLK, DPAGT1, DPM1, DPM2, DPM3, DYNC1H1, DYSF, ECEL1, EMD, ENO3, ERBB3, ETFA, ETFB, ETFDH, FAM111B, FBN2, FBXO32, FHL1, FHL2, FKBP14, FKRP, FKTN, FLNC, GATM, GBE1, GFPT1, GMPPB, GNB4, GNE, GOSR2, GTDC2, GYG1, GYS1, GAA, HSPB8, HSPG2, IGHMBP2, ISCU, ISPD, ITGA7, ITPR1, KBTBD13, KCNE1, KCNE3, KCNJ2, KCNQ1, KLHL40, KLHL41, KLHL9, LAMA2, LAMB1, LAMB2, LAMP1, LAMP2, LARGE, LDB3, LDHA, LDHB, LIMS2, LMNA, LMOD3, LPIN1, LRP4, MATR3, MBNL1, MEGF10, MGME1, MICU1, MSTN, MTM1, MTMR14, MTTP, MUSK, MYBPC1, MYBPC2, MYBPC3, MYF6, MYH14, MYH2, MYH3, MYH4, MYH7, MYH8, MYL1, MYL3, MYLPF, MYOM1, MYOM2, MYOM3, MYOT, MYOZ1, MYOZ2, MYOZ3, MYPN, NBR1, NEB, NEBL, NEFL, NTRK1, OBSL1, OPA1, ORAI1, PABPN1, PDK3, PDLIM3, PDLIM5, PDLIM7, PFKM, PGAM2, PGK1, PGM1, PHKA1, PIP5K1C, PLEC, PLEKHG4, LEKHG5, PLN, PNPLA2, POLG, POLG2, POMGNT1, POMK, POMT1, POMT2, PREPL, PRKAG2, PTPLA, PTRF, PYGM, RAPSN, RBCK1, RRM2B, RYR1, SCN4A, SEPN1, SGCA, SGCB, SGCD, SGCG, SIL1, SLC22A5, SLC25A20, SLC25A4, SLC52A2, SLC52A3, SLC5A2, SMCHD1, SPEG, QSTM1, SRF, STAC3, STIM1, STIM2, SUCLA2, SYNE1, SYNE2, SYNE3, SYNPO2, TAZ, TCAP, TFG, TIA1, TIAL1, TK2, TMEM43, TMEM5, TMEM55A, TMOD3, TNNC1, TNNC2, TNNI1, TNNI2, NNI3, TNNT1, TNNT3, TNPO3, TNXB, TOR1AIP1, TPM1, TPM2, TPM3, TPP1, TRAPPC1, TRAPPC11, TRIM32, TRIM54, TRIM55, TRIM63, TRPV4, TTC19, TTN, TTR, UBA1, VAPB, VCP, VIPAS39, VMA21, VRK1, YARS2.
**Genes associated with congenital nemaline myopathy**:
ACTA1, CFL2, KBTBD13, KLH40, KLH41,LMOD3, MYPN, NEB, NEBL,TNNT1, TNNT3, TPM1, TPM2, TPM3

**Fig. 3:**
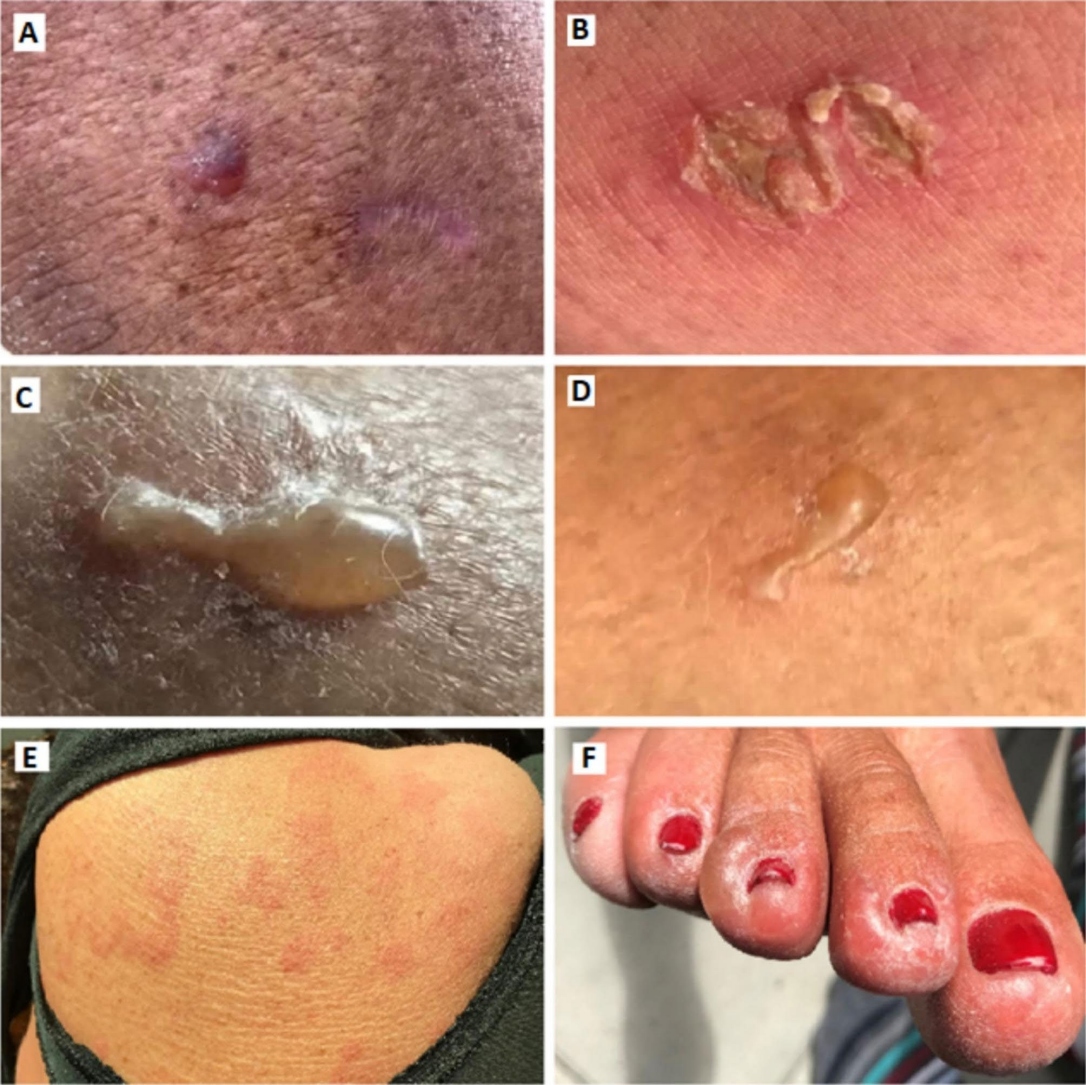
Skin manifestations. **A-F.** Skin manifestations with rashes and blisters on the trunk, proximal and distal extremities

The skin lesions were present parallel with the patient’s neuromuscular symptoms. Figure 3 A-F. Repeated biopsies of the skin lesions could not reveal any explanatory underlying dermatological condition. One biopsy showed changes consistent with urticaria, as expected; the rest showed ulceration, granulomatous reaction, and oedema of the papillary dermis with perivascular infiltration of lymphocytes. Examination for herpes virus infection was negative. An unspecific immune reaction with immunoglobulin A (IgA) and immunoglobulin M (IgM) deposit on the surface of the endothelium of the blood vessels was detected in one out of three biopsies.

Cardiological and pulmonary examinations were also carried out. Pulmonary function tests were normal. The cardiac workup was without pathology aside from slight left ventricular hypertrophy related to the patient`s hypertension. Ejection fraction was 60%, no signs of cardiomyopathy were observed.

## Treatment

**Fig. 4:**
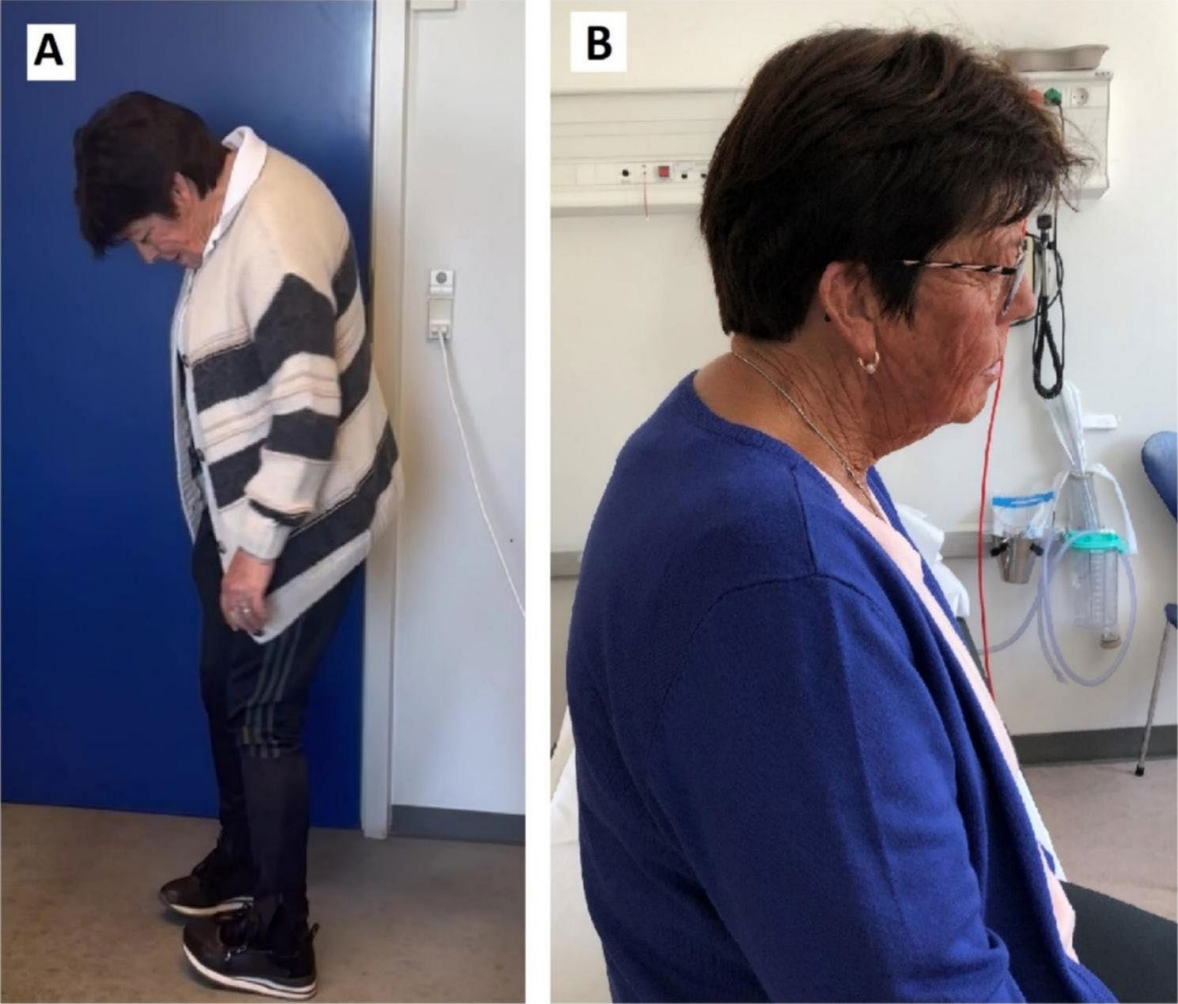
Pictures of the patient showing the head drop. **A**. Before treatment **B**. After treatment

Pyridostigmine treatment was introduced early during the workup because of the neuromuscular transmission defect seen on single fiber EMG and the residual thymus detected with the CT scans. The dose was titrated slowly up to 360 mg daily (6 × 60 mg), without a significant effect. Conversely, the patient experienced diarrhea despite atropine treatment, thus pyridostigmine administration was stopped. Afterwards the patient received steroids over a period of 4 months. The maximum dose was 60 mg/day, calculated after weight (1 mg/kg). The dose was tapered gradually. The patient experienced amelioration of the symptoms while on doses higher than 40 mg. Neck extension muscle strength increased to 4 on the MRC scale and dysphagia decreased. However, side-effects as oedema, worsening of the previously known hypertension and significant psychotic symptoms appeared. Therefore, steroids had to be discontinued. The patient`s therapy was changed to intravenous immunoglobulin (IVIG), which has been administered for over 3 years. The standard dose of 2 g/kg has been used (120 g calculated after a weight of 60 kg) and given over 5 days every 5–6 weeks. The patient experienced a prompt response after the first cycle, neck extension muscle strength increased from 3 to 4 on the MRC scale, dysphagia disappeared. Figure 4. The mild ptosis also improved.

The presentation of skin symptoms was variable depending on the immunosuppressive treatment. A significant improvement was seen after the start of intravenous immunoglobulin therapy with slight fluctuations depending on the weeks elapsed after the last treatment. The patient received IVIG every 5–6 weeks and slight worsening was seen 1–2 weeks before each treatment in the number of blisters.

## Discussion and conclusions

The ultrastructural presence of nemaline rods in the muscle fibers and the characteristically late appearance of symptoms suggested SLONM. Clinical evaluation showed paraspinal, neck-extensor weakness, which presented first, later accompanied by bilateral ptosis and dysphagia, corresponding to typical symptoms seen in larger cohorts [2] [4]. Upper and lower extremity involvement, described recently amongst SLONM patients with a slow, subacute presentation [6], could not be detected.

Muscle biopsy showed round and angular atrophic fibers with type 1 fiber predominance. The core feature of nemaline rods was exclusively observed in the atrophic fibers, contrary to congenital nemaline myopathies where an abundance of nemaline rods can be seen in non-atrophic fibers as well [6] [7]. Intranuclear rods could not be observed. Nemaline rods have been described in other muscle diseases as well [8], however these had been excluded with the extensive genetic, biochemical, and radiological investigations during the diagnostic workup.

Mitochondrial changes like ragged-red and COX negative fibers are frequent abnormalities observed in SLONM [2] [4]. These were accompanied by abnormal mitochondria in our case. Etiology of the mitochondrial pathology is still unknow in SLONM, although the effect of Z-disc remodeling on skeletal muscle fiber mitochondria has been suggested [2].

An immune-mediated etiology has been implicated in SLONM [2] [3] [4] [5] [9]. Inflammatory features such as CD68 positive macrophage infiltration, mild MHC expression have been reported without the presence of lymphocytes [2] [4]. An association has been observed between the nemaline rod containing fibers and the inflammatory changes. A recent study compared muscle biopsies from MGUS related SLONM cases with those without and concluded that the two entities are not inherently different from each other histologically [4]. In both types of SLONM, signs of mild inflammation were found frequently, although cases without inflammation have also been described [4]. Another study showed evidence for upregulation of immune-related proteins despite the lack of inflammation on muscle biopsy in SLONM [9], suggesting that immune-related processes are involved in all cases, but to various extent. MGUS could be a sign for a more aggressive immune response [9].

In our case the patient’s muscle biopsy did not show distinct inflammatory changes. However, this alone does not imply that response to immunosuppressive treatment can be excluded. Various reports on SLONM patients without histological signs of inflammation have described different responses to immunosuppressive therapy. MGUS SLONM patients without signs of inflammation benefitted from over a 24 months-treatment with IVIG [4] [5] [10], while stabilization could be achieved in a patient non-MGUS SLONM [4] [7]. In fact, our patient also responded to immunosuppressive therapy with a significant improvement in neck-extensor muscle strength and dysphagia. The initial part of the treatment regime were steroids, but these had to be discontinued, because the patient developed psychosis as a side effect. Therefore, treatment was changed to continuous IVIG therapy.

The presentation of myopathic symptoms related to SLONM preceded by blistering skin manifestations is a new constellation. The presence of two different disorders cannot be excluded, however a connection between the muscle and the skin presentations can also be considered. The positive effect of the immunosuppressive treatment regarding both manifestations can essentially be explained by a possible, common, immunogenic cause. Dermatological symptoms have been described in other neuromuscular diseases and in SLONM as well [11]. However, the cases described in relation to SLONM have been observed in the context of another autoimmune comorbidity, for example SLE [12], or Sjögren [13] syndrome. In these cases, characteristic clinical symptoms and biochemical data supported the above-mentioned conditions. In connection with our patient, investigations regarding a specific underlying autoimmune disease did not yield positive results. Nevertheless, skin biopsies showed subtle immune reactions, suggesting an autoimmune component in the pathology. Furthermore, the patient’s dermatological symptoms improved after immunotherapy.

The presentation of myasthenia gravis (MG) with SLONM has been documented before [14] [15]. However, in our case, characteristic serological signs of MG could not be found, and the clinical presentation was atypical, without daily variation of symptoms and fatiguability. There was also a lack of effect of pyridostigmine administration. The relevance of the residual thymus with the histology of thymic follicular hyperplasia is difficult to determine. The additional findings of signs of neuromuscular transmission defect seen with the sfEMG can, however, raise the possibility of a comorbidity with seronegative myasthenia gravis. Nevertheless, it is also feasible, that the described sfEMG changes are secondary to myopathy, which has been documented before.

Our case describes an unusual presentation of SLONM in a non-HIV, non-MGUS subtype with concurrent dermatological symptoms, where immunosuppressive therapy was effective for both muscle and skin manifestations. Thus, our data can provide further support for an immune pathogenesis in sporadic late onset nemaline myopathy.

## Data Availability

All data generated or analyzed during this study are included in this published article.

## Abbreviations

SLONM: Sporadic late onset nemaline myopathy
MGUS: monoclonal gammopathy of unknown significance
HIV: human immunodeficiency virus
MRC: Medical Research Council
LDH: lactate dehydrogenase
EMG: electromyography
sfEMG: single fiber electromyography
COX: cytochrome c oxidase
NGS: next generation sequencing
IgA: immunoglobulin A
IgM: immunoglobulin M
MG: myasthenia gravis
IVIG: intravenous immunoglobulin
MHC: class I human major histocompatibility complex
